# The Attitude of Great Writers towards Disease

**Published:** 1892-07-02

**Authors:** 


### The Attitude of Great Writers Towards Disease.

### IV.—JANE AUSTEN.

The habits of the generation among which Jane Austen is numbered, inclined to a Spartan self-control which in the present day it is hard to realise. Especially was this the case in the country, where rigid simplicity and regularity of life led to a tone of thought which condemned even the slightest approach to self-indulgence. One of the last of the stock, an old lady of ninety, was heard not long ago to reprove her niece (over fifty) for the crime of sitting in an easy-chair, "she did not like to see young people lounging." Illness alone was considered sufficient excuse for relaxing ordinary habits, and even this was not always accepted, so great was the dread of appearing selfish or "particular."

The scanty record which we have of Jane Austen's life shows her conforming in mind and body to these theories of discipline. Light ailments are ignored or laughed away. Severe illness is an exercise for quiet submission and effacement of self. We see her in her last illness reclining uneasily on three chairs rather than use the sofa, lest her mother might scruple to be on it as much as was good for her. The sweet and wholesome sunshine of her wit played even over her last sufferings, and but two months before her death she could write to her nephew, "Mr. Lyford says he will cure me, and if he fails I shall draw up a memorial and lay it before the Dean and Chapter, and have no doubt of redress from that pious, learned, and disinterested body."

Bearing in mind these traits of character in the authoress, it is easier to understand her attitude towards the invalids of her novels. Among all the various humours which delighted her among her acquaintance nothing seems to have stirred her amusement so much as their manner of petting their little indispositions. There was, perhaps, some encouragement for "malingering" in days when being ill was the only lawful road to being comfortable, and this may account for the constant presence of the valetudinarian is those little groups of country society created by Miss Austen's genius. Yet if the theme recurs more than once, it is relieved from any suspicion of repetition by the continual variety and freshness in the development of the characters, which preserve them from sameness in themselves and mark them out as individuals, not types. We all know the familiar round of the book invalid; the irritable, the plaintive, the saintly. Wearisome people at best, and sadly alike. But Mr. Woodhouse could never weary us. Emma's spirits and patience may give way sometimes, but to the reader his gentle talk and his little kindly selfish ways lend enjoyment as fresh in the twentieth as in the first perusal. His unexpected touches of petulance, his warmth in defence of Perry, his confidence in his cook and coachman, his deference to brides and enmity to marriage; the little traits are endless and continually new. Compare him, too, with his daughter, Mrs. John Knightley. With all her father's interest in her own and her neighbour's health, with equal dependence on her pet doctor, and with the strong family likeness revealed in the very turn of her phrases, how far she is from a mere counterpart. Who but Miss Austen could introduce and contrast two such characters where the contrast depends on such delicate points as would not easily be apprehended in real life, and which yet lend a pervading charm to both father and daughter. They are at their best together on the first evening of Mrs. Knightley's visit home.

"My poor, dear Isabella, how long it is, how terribly long since you were here! And how tired you must be after your journey! You must go to bed early, my dear, and I recommend a little gruel to you before you go. You and I will have a nice basin of gruel together. My dear Emma, suppose we all have a little gruel." The gruel is ordered, and the conversation turns upon the neighbours.

"Good old Mrs. Bates! I will call upon her to-morrow and take my children. They are always so pleased to see my children. And that excellent Miss Bates—such thorough worthy people. How are they, sir?"

"Why, pretty well, my dear, upon the whole. But poor Mrs. Bates had a bad cold about a month ago."

"How sorry I am, but colds were never so prevalent as they have been this autumn. Mr. Wingfield told me that he has never known them more general or heavy, except when it had been quite an influenza."

"That has been a good deal the case, my dear, but not to the degree you mention. Perry says that colds have been very general, but not so heavy as he has very often known them in November. Perry does not call it altogether a sickly season."

"No, I do not know that Mr. Wingfield considers it very sickly, except ——"

"Ah, my poor dear child, the truth is that in London it is always a sickly season. Nobody is healthy in London, nobody can be. It is a dreadful thing to have you forced to live there—so far off—and the air so bad."

"No, indeed, *we* are not at all in a bad air. Our part of London is so very superior to most others. We are so very airy."

It is hard to turn away from the pair. But this skill in enlisting our sympathies for the victims of her satire; her entire escape from all touch of hardness or ill-nature is very remarkable in Miss Austen. To be continually laughing at her neighbours, yet to love them through it all, is her gift alone in fiction. This is the clue to the puzzle that her bores are better company than other people's wits, and her self-absorbed invalids become warm personal friends, while the heroines of the modern novel lie forgotten on the shelf. It is as though her gratitude towards those who unconsciously provided her with so much entertainment engendered in her a real liking for their eccentricities. How she liked to hear Mr. Woodhouse worry his friends about their health. "Young ladies are delicate plants. They should take care of their health and their complexion. My dear, did you change your stockings?" And in the thrilling moment of Lydia's elopement, when her fate is still unsettled, the authoress finds time to join Mr. Bennet in enjoying his wife's nerves. "Above all things keep Mr. Bennet from fighting. Tell him what a dreadful state I am in—that I am frightened out of my wits—I have such tremblings, such flutterings, all over me, such spasms in my side, and pains in my head, and such beatings at heart that I can get no rest by night nor by day."

Even Mrs. Charles Musgrove, whose selfishness is almost too apparent, has her pleasing moments. Does not everyone number her among his acquaintances? "Always very ill—a great deal worse than she ever owns," and whose "sore throats, you know, are always worse than anybody's."

The unnamed and unfinished fragment upon which Miss Austen was engaged at the time of her death, furnishes two more striking portraits in Miss Diana Parker and her sister, who "must be always either very busy for the good of others, or else extremely ill themselves." "Two years ago I happened to be calling on Mrs. Sheldon, when her coachman sprained his foot, and could hardly limp into the house; but by the immediate use of friction alone (I rubbed his ancle with my own hands for four hours without intermission) he was well in three days." And again, "Susan has been suffering much from headache, and six leeches a day, for ten days together, relieved her so little that we thought it right to change our measures; she has accordingly had three teeth drawn, and is decidedly better; but her nerves are a good deal deranged, she can only speak in a whisper, and fainted away this morning on poor Arthur's trying to suppress a cough."

It would be unfair to suppose that Miss Austen has no feeling for real illness. Two, at least of her heroines, the uncommunicative Jane Fairfax and pretty Fanny Price, are gifted with a very interesting delicacy of constitution, and the heroism of the crippled Mrs. Smith in "Persuasion" has its full need of appreciation. Both in the authoress and in her characters a quick sympathy with real grief and suffering are linked with that irresistible laugh at the pretence of either. The one quality is but the completion of the other in her philosophy of life—gaily ironical towards the human and ridiculous; very tender and reverent towards all which reaches forward to the Divine.

